# A Crowd-Sourced Database of Coronamusic: Documenting Online Making and Sharing of Music During the COVID-19 Pandemic

**DOI:** 10.3389/fpsyg.2021.684083

**Published:** 2021-06-18

**Authors:** Niels Chr. Hansen, John Melvin G. Treider, Dana Swarbrick, Joshua S. Bamford, Johanna Wilson, Jonna Katariina Vuoskoski

**Affiliations:** ^1^Aarhus Institute of Advanced Studies, Aarhus University, Aarhus, Denmark; ^2^Department of Clinical Medicine, Center for Music in the Brain, Aarhus University and Royal Academy of Music Aarhus/Aalborg, Aarhus, Denmark; ^3^Department of Musicology & Department of Psychology, RITMO Centre for Interdisciplinary Studies in Rhythm, Time and Movement, University of Oslo, Oslo, Norway; ^4^Social Body Lab, Institute of Cognitive and Evolutionary Anthropology, School of Anthropology & Museum Ethnography, University of Oxford, Oxford, United Kingdom; ^5^School of Psychological Science, The University of Western Australia, Perth, WA, Australia; ^6^Department of Music, Arts and Culture Studies, Centre for Interdisciplinary Music Research, University of Jyväskylä, Jyväskylä, Finland

**Keywords:** music, COVID-19, crowdsourcing, emotion, video, corpus, social media, YouTube

## Introduction

When a sweeping COVID-19 pandemic forced cultural venues, schools, and social hangouts into hibernation in early 2020, music life relocated to the digital world. On social media platforms like YouTube, Facebook, Twitter, and TikTok, sofas and balconies took center stage for musical performances presented as live-streamed concerts and recorded videos. Amateurs and professional musicians alike embraced digital formats and innovated novel genres of corona-themed music. Adapting the well-known “musicking” term from cultural musicology (Small, 1999), we will characterize the diverse practices of listening to, playing, dancing to, composing, rehearsing, improvising, discussing, exploring, and innovating musical products during lockdown with explicit or implicit reference to the novel coronavirus and/or pandemic life circumstances as “corona-musicking.” By extension, audiovisual products of such corona-musicking behavior will be defined collectively as “coronamusic.” To best facilitate future work, these definitions are intentionally broad and minimally exclusive (cf. Small, 1999). This study aims to establish the CORONAMUSIC DATABASE–a crowdsourced corpus of links to coronamusic videos and news media reports (https://osf.io/y7z28/). This constitutes the first readily accessible and searchable resource for researchers from all disciplines with an interest in documenting and investigating the musical dynamics underlying the pandemic.

Because establishing a typology of coronamusic lies beyond the scope of a data report, we will restrict ourselves to mentioning examples here. Across the globe, topical tunes were composed, and humorous COVID-19 lyrics were invented for well-known songs, resulting in thought-provoking contrafacta some of which were designed to process an unfamiliar everyday life in safe sonic environments. Reminiscent of previous outbreaks such as “The Plague of Saint Charles” hitting Milan in 1576 (Chiu, 2020), daily routines involving balcony singing and joint, steadfast clapping for healthcare workers accompanied by loud vocalizations, whistling, and percussive use of kitchen utensils (Taylor, 2020) offered musical ways of imposing structure upon chaos and expressing gratitude through ritualistic behavior (Imber-Black, 2020). When music teaching moved online (Philippe et al., 2020; de Bruin, 2021), musical skills and social cohesion were nurtured through lockdown-compatible, video-conference-based rehearsal formats (Datta, 2020; Daffern et al., 2021; MacDonald et al., 2021). Governmental and private organizations commissioned and disseminated health information videos promoting hand-washing (Thampi et al., 2020) and similar protective measures through musical mnemonics and dance challenges. Deprived of opportunities for live concert attendance and clubbing, some people participated in virtual raves (Palamar and Acosta, 2020). Corona-musicking behaviors were documented through splitscreen videos flooding the social media landscape under hashtags like #coronasongs, #quarantunes, #covidance, #pandemix, and #songsofcomfort. The diversity and complexity of coronamusic repertoire call for descriptive research efforts with an epistemologically open mindset, ideally liberated from traditional disciplinary boundaries.

An important question concerns when and how much people engaged with music during lockdown. Music listening through audio-based streaming platforms (e.g., Spotify) decreased considerably, most likely due to changes in music-accompanied behaviors like public commuting and gym exercise (Sim et al., 2020). The same report found dramatic surges in video-based services (e.g., YouTube). Some platform-neutral, self-report measures indeed suggest increased music listening during lockdown (Cabedo-Mas et al., 2020; Mas-Herrero et al., 2020; Fink et al., 2021; see, however, Krause et al., 2020). Qualitative findings support these lifestyle-related changes in music consumption, including the switch to video-based streaming (Carlson et al., 2021). While the trend of creating and sharing corona-themed musical content persisted throughout 2020, corona-musicking may have been most prominent during the early months of the pandemic (i.e., March–May). This would be consistent with findings that music listening may have decreased slightly about two months after lockdown began (Krause et al., 2020). Despite these objective and subjective reports of pandemic musicking at home, empirical data on online coronamusic sharing remain scarce.

Scientific knowledge on intrinsic motivations for pursuing corona-musicking is gaining territory. Across 11 countries, Granot et al. (2021) found that musical engagement provided the most effective, physically distanced leisure activity for achieving well-being goals. Mas-Herrero et al. (2020) showed that people in Italy, Spain, and USA picked music-related activities over entertainment (e.g., watching series, movies, reading books) and physical exercise as the most helpful coping mechanism for pandemic-induced distress. The hours spent on musical activities *during* (but not *before*) lockdown were negatively associated with anxiety and depression, and this effect was mediated by individual reward sensitivity. Other reports indicate that nostalgic music listening—which is known to induce positive emotions toward one's own autobiographical memories in general (Sugimori et al., 2020) and during lockdown (Gibbs and Egermann, 2021)—was especially prominent (Yeung, 2020). Greatest repertoire specificity was achieved by another large-scale, multi-country study by Fink et al. (2021) whose results assigned particular significance to corona-themed music. Specifically one's interest in how other people used coronamusic to respond to the crisis most strongly predicted how much musical engagement assisted emotional and social coping. Consistent with the social surrogacy theory (Schäfer and Eerola, 2020), people experiencing positive emotions used music listening and making as a proxy for social interaction. Relatedly, others have speculated that sharing emotions through music has enhanced our ability to tolerate pandemic-induced, transient uncertainty states (Sarasso et al., 2021).

Given the humorous nature and lockdown-related topics of much coronamusic, these recent findings seem consistent with research on motivations for media use more broadly. Eden et al. (2020) found that pandemic-induced anxiety was associated with greater media use overall with a particular prominence of content designed for reframing pertinent stressors pursued with an eudaimonic motivation to tackle existential questions of purpose in life and moral values. Approach coping behaviors and related motivations were in turn associated with positive emotions and psychological flourishing. Tackling anxiety through humoristic coping was, furthermore, associated with good mental health.

In addition to social media and fiction, people heavily consumed news during lockdown (Fink et al., 2021). A non-negligible proportion of COVID-19-related news dealt with aspects of coronamusicking, thus making news media informative for a comprehensive understanding of this phenomenon. A recent report from *European Parliamentary Research Service* describes how today's media outlets are subject to growing commercial pressure acting on a globalized playing field where information spreads at unseen speeds and competition for attention is fierce (Fletcher and Jenkins, 2019). The resulting breaking news culture leaves limited space for critical fact-checking and promotes sensationalist journalism and self-fulfilling filter bubbles entailing an imminent risk of accidental dissemination of insufficiently verified information; these tendencies were only exacerbated during the pandemic (Ali, 2020). Anecdotal observations reveal that much media coverage of coronamusic initiatives emphasized positive emotions like joy, humor, and togetherness (Taylor, 2020). This potential positive affective bias contrasts starkly with the predominantly negative sentiments dominating other areas of the public sphere (Chandrasekaran et al., 2020; Li et al., 2020). This invites nuanced empirical substantiation for topics and sentiment in coronamusic-themed news coverage. Therefore, in addition to COVID-19-themed music videos from social media, a separate subset comprising news media coverage of coronamusicking was included in the database.

In light of coronamusic's novelty and significance in the everyday lives and psychological coping behaviors of the global population, a corona-themed musical data resource designed to enable scientific study is needed. Despite the release of large datasets surveying psychological well-being (e.g., Yamada et al., 2021) and media use (e.g., reading habits: Salmerón et al., 2020), to our knowledge, no public corpora document musical content used and produced during the societal lockdowns of 2020. Several desirable features spring to mind. First, while many prior music video-related datasets have been text corpora comprising YouTube user comments (e.g., Uryupina et al., 2014; Bassignana et al., 2020; see, however, Osborn et al., 2020), the CORONAMUSIC DATABASE contains links to the videos themselves. Second, to facilitate capturing the diversity in cultures, sentiments, musical genres, and instrumentation, crowdsourcing was used (Hale et al., 2021). However, because sampling relied on personal networks and because all gathered data needed to be interpretable and codable by our own research team–with all its inherent cultural biases–an emphasis on Europe and the English-speaking world (i.e., USA, Canada, Australia, New Zealand) was unavoidable. Finally, to ensure a sizable corpus, real-time crowdsourcing was supplemented with systematic retrospective sampling.

## Methods

A database of links to COVID-19-themed music videos and news media content representative of the coronamusic phenomenon was created via crowdsourcing (Section Sampling). The video-based subset was subsequently extended with retrospectively sampled YouTube videos to ensure a substantial daily representation for the first two lockdown months. While audio-only content (e.g., SoundCloud) was permitted, we focused on videos because music consumption partly migrated from audio- to video-based platforms during COVID-19 lockdown (Sim et al., 2020; Carlson et al., 2021). During recent years, music videos have been the top-ranking content genre on YouTube (Liikkanen and Salovaara, 2015), which is optimized for user content creation and has become the most popular music streaming service in some countries (Sohn, 2018). All data entries were systematically coded by the research team to enhance searchability (Section Coding).

### Sampling

#### Crowdsourced Sampling

On 26th March 2020, an openly accessible Google Sheet was created inviting the public to supply URL links to videos, media coverage, hashtags, online concerts, and other resources in any language deemed to exemplify how music was used during the novel coronavirus pandemic. On 3rd April 2020, an online survey was launched on the SurveyXact platform (Rambøll Management Consulting, Denmark) to facilitate content collection.

To estimate the reach of recruitment efforts, statistics were sourced from Twitter. Specifically, tweets from NH personal account included an initial announcement of the Google Sheet with a clarifying follow-up tweet on 26 March, an announcement of the SurveyXact questionnaire with two follow-up tweets on 3 April, a follow-up announcement in Danish on 20 April, and a closing notice on 23 July. A total of 30,242 impressions (i.e., number of views by anyone), 1,033 engagements (i.e., number of interactions generated), 118 retweets, and 113 likes were obtained. Further unquantified activity is expected from retweets and shares on other platforms (e.g., Facebook). Additionally, all contributors were encouraged to share survey links within their own network, and the database was advertised to research colleagues at virtual launching events for the MUSICOVID network with >250 global attendance on 19 May. Finally, the overall research project was promoted with survey links on the researchers' university webpages and by national news media in Denmark, Norway, Finland, Sweden, and The Netherlands.

#### Retrospective Sampling

**Figure 1:**
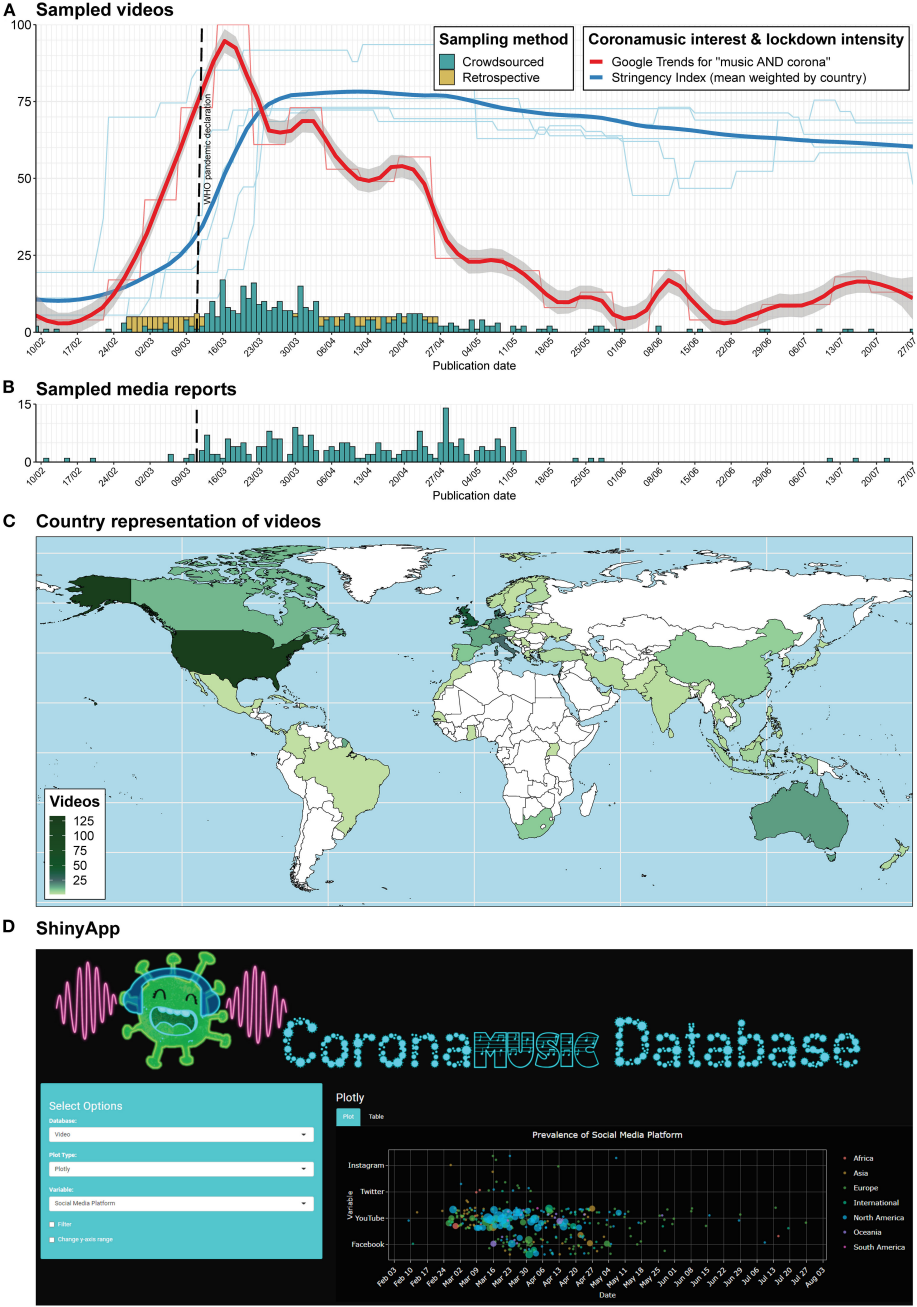
Stacked histograms depicting crowdsourced (turquoise) and retrospectively sampled (yellow) **(A)** music video and **(B)** news media content in relation to the time course of coronamusic interest and lockdown intensity in February–July 2020. Coronamusic interest was quantified via weekly Google Trends data for the search term “music AND corona” (thin red line, sourced on 12 February 2021). The thick red line is a LOESS curve (locally estimated scatterplot smoothing) fitted to weekly Google Trends data copied across all days of the week. Lockdown intensity was quantified via the stringency index from *Oxford COVID-19 Government Response Tracker* (Hale et al., 2021). The thin blue lines represent country-specific stringency indices for USA, UK, Denmark, Italy, and Australia. These countries ranked amongst the most prevalent countries–represented with ≥15 videos–in the crowdsourced proportion of the database and were used as geolocation specifiers for the Google searches underlying retrospective sampling (cf. Section Retrospective sampling). The thick blue line represents a LOESS curve fitted to the means of stringency indices weighted by the number of videos across all countries represented in the crowdsourced proportion of the video database. The scale of the y-axis simultaneously indicates the number of videos (histograms), percentage of maximum search activity for Google trends throughout 2020 (red lines), and stringency index (blue lines). The outbreak of SARS-CoV-2 virus was declared a pandemic by the World Health Organization on 11 March 2020 (dashed black line). Note that the retrospectively sampled range from 27 February to 26 April covers this pandemic declaration date along with all dates where Google trends exceeded 25% of the maximum for 2020, and dates representative of the peak of lockdown measures for the countries most prominently featured in the database. **(C)** World map of countries represented in the video database colored according to prevalence. **(D)** Example screenshot from the associated online ShinyApp where users can explore the database content on their own. Logo includes images of headphones provided by Mozilla, cute coronavirus by Manuela Molina (@mindheart.kids), and coronaviral font, all licensed under CC BY 4.0.

Crowdsourced videos and media reports had the highest representation during March-April 2020 (Figures 1A,B). This coincided reasonably well-both with the peak in the use of “music AND corona” as a Google search term (sourced on 12 February 2021) and the upsurge in lockdown restrictions across the represented countries (Figure 1A). Yet, to facilitate future longitudinal studies, further YouTube videos were sampled retrospectively ensuring a minimum of five items per calendar date during the initial two lockdown months.

Retrospective sampling employed the “googlesearch” library in Python (script available on OSF: https://osf.io/y7z28/). We decided to reproduce the cultural biases of the original dataset by focusing on countries represented with ≥15 crowdsourced videos (USA: 133; UK: 42; Denmark: 32; Israel: 27; Italy: 25; Australia: 15),^1^ but excluded Israel due to lacking Hebrew coding competence. The first day with non-zero stringency indices across all five countries (27 February) was chosen as starting date. Because Google does not permit single-day searches and requires country/region specification, overlapping two-day windows were used, and the five target countries were included as search locations one by one (using the “gl” tag). For each window, 25 candidate URLs were sourced for each country. For 27 February, for example, the following search term was applied (adhering to the current upper word limit for Google searches):

site:youtube.com before:2020-02-28 after:2020-02-27 (music OR musica OR musik OR musique OR muziek OR song OR tune OR lied OR liedje OR chanson OR canzone OR sang) AND (covid OR covid19 OR “covid-19” OR coronavirus OR corona OR “sars-cov-2” OR #quarantunes OR #covidance OR #coronasongs OR #coronamusic OR #TogetherAtHome OR #StayHome OR #Pandemix OR #COVered19 OR #gratitunes)

Thus, a music-related term was required in combination with a coronavirus-themed term or one of the nine most common crowdsourced hashtags. In addition to the target-country languages, Dutch, German, and French terms were included as these languages were represented with ≥10 crowdsourced videos from Netherlands (14), Germany (15), France (13), and Canada (11) (Figure 1C). The Python script was run from an HP machine (Windows 10) in Oslo (i.e., neutral territory outside the five target countries), on 31 October at 8:00 CET and concluded ~24 hours later.

To produce a single prioritized list of candidate URLs for each date, rows were selected from the top of the generated output using a randomly drawn vector of country labels weighted according to the crowdsourced country distribution while avoiding duplicate URLs.

### Coding

#### Videos

For characterizing and classifying videos, a coding scheme was developed. Specifically, 15 videos were first randomly selected for exploratory content analysis by all authors independently. Codes based on *ad-hoc* categories from Google Sheet/SurveyXact were subsequently compared to check for inconsistencies, add and merge categories, and refine definitions when necessary.

The final coding scheme agreed upon by all team members comprised:

- *****ID***:** Arbitrarily assigned unique indices.
- *****Category***:** Sub-corpus (i.e., video, media, other).
- *****Source***:** Sampling origin (i.e., GoogleSheet, SurveyXact, Retrospective)
- *****URLs***:** URL(s) locating the item.
- *****Title***:** Title sourced online or submitted by contributor.
- *****PublicationDate***:** Date stamp for publication.
- *****SoMePlatform***:** Social media platform (e.g., YouTube, Facebook, Twitter, TikTok, Instagram, SoundCloud, Vimeo).
- JointMusicking^*^: Whether >1 person is visibly performing together.
- OrigCovidSong^*^: Whether the performer(s) have composed the music themselves during the coronavirus crisis (regardless whether the lyrics were unambiguously COVID-related).
- OrigCovidLyrics^*^: Whether lyrics make reference to the coronavirus situation.
- Movement^*^: Whether movement is featured and/or encouraged in the video, including dancing, yoga, and prominent moving around while performing, to an extent beyond what is minimally required for successful musical performance/attendance.
- HealthInfo^*^: Whether instructions like “stay home,” “wash hands,” “wear a mask” occur.
- Conflict^*^: Whether content (i.e., lyrics, visual gestures, spoken utterances, musical features) refers to conflict–e.g., between nations, fellow citizens, or cohabitants.
- *****Country***:** Performers' nationality and/or residence country.
- *****LyricsLanguage***:** Language(s) of lyrics.
- Setting^**^: Setting(s) of music making/visual content (e.g., outside, home, kitchen, balcony, venue).
- Genre^**^: Musical genre(s) represented, using at least one of the 23 categories from *Revised Short Test of Music Preferences* (STOMP-R; Rentfrow and Gosling, 2003) along with voluntary free-text labels.
- Emotions^**^: Emotions represented or evoked. Free-text responses supplemented nine prompts generated through author consensus (anger, being moved, gratitude, grief/sadness, happiness, humor, loneliness, togetherness). Consensus was reached during coding scheme development and was informed by the authors' continual watching of coronamusic videos during the pandemic.
- Features^**^: All-inclusive category for any other characteristic features (e.g., splitscreen, for kids, balcony performance, patriotism).

There were three variable types: (a) *factual variables* (no asterisk), (b) *binary variables* (single asterisk), and (c) *open-coded variables* (double asterisks). Whereas binary variables were dichotomous, open-coded variables used inclusive coding allowing for multiple values and NAs without enforcing mutual exclusivity (e.g., “home” and “kitchen” settings co-existed for the same item). Two further variables indicated date (RetroSampleDate) and geolocation (RetroSampleCountry) for retrospective sampling. “Features” labels were developed and refined during group meetings throughout the coding process as more videos were viewed (and re-coded when necessary).

All video URLs were randomly assigned a first and second coder from amongst the authors (see FirstCoder and SecondCoder columns). After all coders had independently coded their assigned videos, conflicts for binary variables were resolved during bilateral virtual meetings whereas open-coded variables were combined across coders. A small subset of unresolved conflicts and principled issues were determined *in plenum*. This systematic procedure ensured that the vast majority of factual and binary codings achieved 100% inter-rater agreement between two coders whereas the remaining more ambiguous codings always had support from at least three authors.

Final editorial proofreading and streamlining was performed to optimize discoverability; typos were amended, spellings were unified, text was converted to lowercase, nouns were singularized, non-essential URL parameters were removed, and similar categories were merged (e.g., “rehearsal” replaced “choir rehearsal;” “venue” replaced “stage” and “concert hall;” “outside” replaced “outdoors;” “patriotism” replaced “nationalism”). Genre designations constituting parts of STOMP-R categories were re-assigned to these (e.g., “dance/electronica” replaced “dance;” “soul/R&B” replaced “R&B” and “soul”). The project group was consulted when doubt arose.

During coding, duplicates, dysfunctional links, and ineligible submissions were removed, and some videos were re-classified as media or other (see Sections Media and Other (concerts, playlists, etc.). Video compilations curated by news media sources (e.g., with printed text) were only re-classified if a reporter was featured visually or via voice-over. Coronamusic-themed links not including videos or news reports were designated as other. Because codings could differ between items with identical audio but distinct video, those were kept.

#### Media

A coding scheme was developed for news media content using a procedure similar to that described in Section Videos. SoMePlatform, JointMusicking, OrigCovidSong, OrigCovidLyrics, LyricsLanguage, and Genre were excluded due to limited relevance. Country was redefined as “countries of media platform and journalist(s) reporting.” All other video variables were included. Five media-specific variables were added:

- *****MediaPlatform***:** Name of news media source.
- *****MediaFormat***:** Format(s) used (i.e., writtenDigital, writtenPrint, audio, video).
- *****MediaLanguage:***** Language of writing or narrator speech.
- EmbeddedExternalVideo^*^: Whether external video content (e.g., YouTube) is embedded.
- MusicMakerProfessionalism^**^: Professional status of music makers described/depicted (amateur and/or prof).

Media items comprised factual, binary (^*^), and open-ended (^**^) variables and were subject to dual coding with conflict resolution and editorial proofreading as described above. No retrospective sampling was performed.

#### Other (Concerts, Playlists, etc.)

Coronamusic-related items from crowdsourcing whose content met the inclusion criteria for neither video nor media content were assigned to a separate “other” category coded by a single individual, exclusively using factual variables. This third sub-corpus comprised playlists of recorded live-streams and home concerts, free access to digital concert halls and opera houses, online music festivals, virtual choirs/ensembles etc.

## Descriptive Statistics

**Figure 2:**
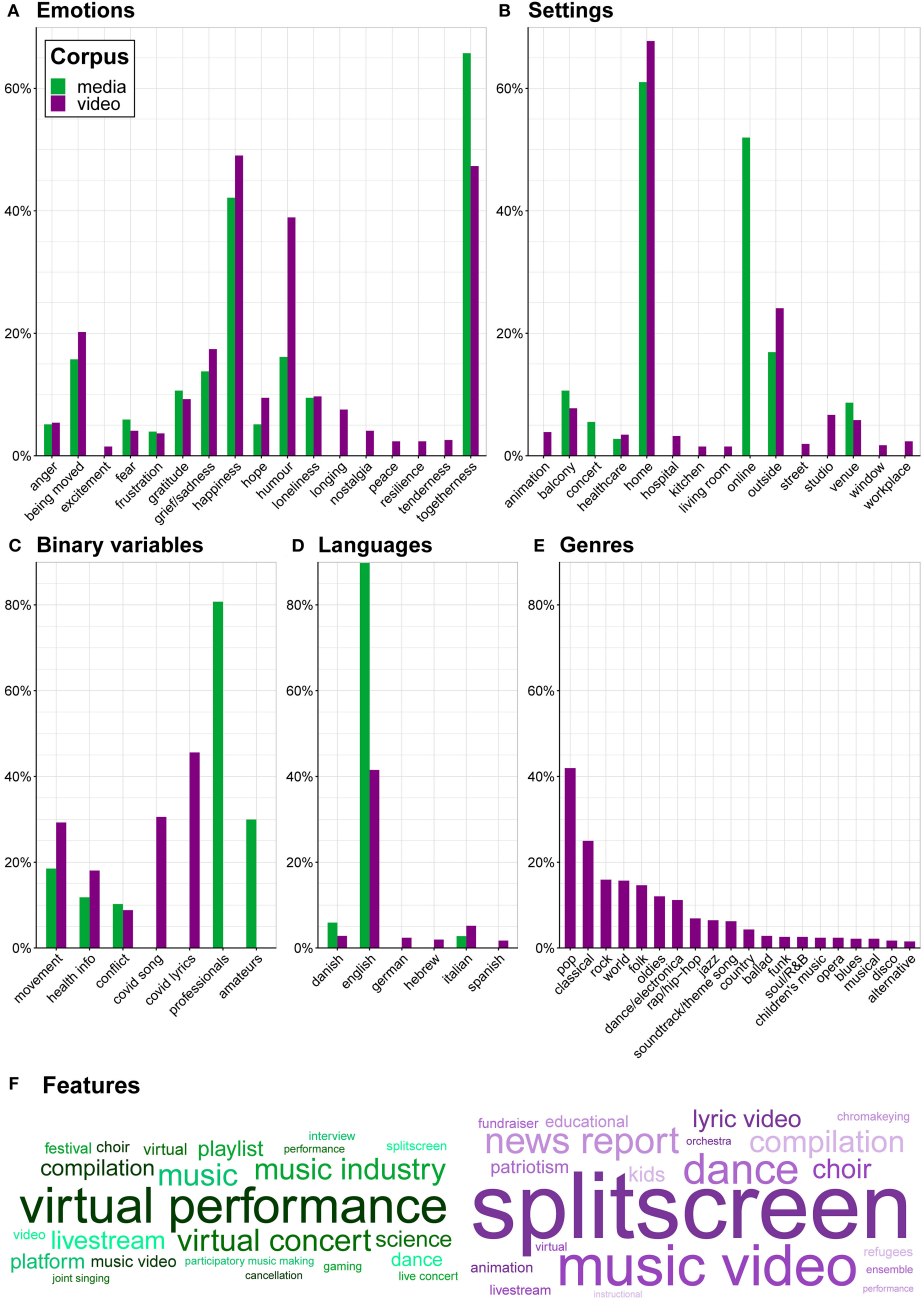
Bar charts for **(A)** emotions, **(B)** settings, **(C)** binary-coded variables, **(D)** languages, and **(E)** musical genres in the media (green) and music video (purple) sub-corpora. The y-axes depict proportion of all music video or media items in which a given term is featured. Because nearly all included variables–except for binary variables in **(C)**–allow for coding of multiple values for one item, summed percentages often exceed 100%. Note that **(D)** refers to language of lyrics in the case of video items and language of journalistic narration in the case of media items. It appears that positive emotions like happiness, humor, togetherness, and being moved were especially prominently displayed and discussed. While the portrayal of the coronamusic phenomenon seems consistent overall between music videos posted on social media and news reports, there is suggestive evidence that sensations of togetherness may have received disproportionally large coverage whereas other positive emotions like happiness and humor—and perhaps especially solitary ones with low arousal and less likelihood of generating inter-personal conflict (e.g., longing, nostalgia, peace, resilience, tenderness)—may have attracted comparatively less media attention. The distribution of musical genres seems largely consistent with those found in a large sample of sales and radio airplay charts in the United States (North et al., 2019); notably, the North-American focus of the comparison corpus may explain comparably lower representation of genres like country, folk, and jazz in the CORONAMUSIC DATABASE. Future research will need to investigate these incidental observations more systematically before any final conclusions can be drawn. **(F)** Word clouds of the Feature variable for media (green) and video (purple) items. The area occupied by each term is proportional to its representation in the relevant sub-corpus. Color nuance is arbitrary, serving solely to enhance legibility.

The final CORONAMUSIC DATABASE contains 465 video items (Google Sheet = 247; SurveyXact = 130; Retrospective = 88), 254 media items (Google Sheet = 197; SurveyXact = 57), and 62 other items (Google Sheet = 57; SurveyXact = 5), published between 8 February and 27 July 2020. All sub-corpora are accessible as separate Excel/CSV files at https://osf.io/y7z28/ and can be explored via our ShinyApp (https://dana-and-monsters.shinyapps.io/corona-music-database/) (Figure 1D). Figure 2 and its accompanying legend provide an overview with tentative observations of key quantitative and qualitative variables.

## Limitations and Future Directions

Sharing music enables people to connect with others (Papinczak et al., 2015). During the COVID-19 lockdown, pandemic-themed, nostalgic, escapist, and humoristic media were used strategically for diverse coping goals (Eden et al., 2020). The CORONAMUSIC DATABASE documents and exemplifies how music sharing on social media may have contributed to these media types.

This corpus suffers from methodological limitations. First, sampling was biased toward wealthy, industrialized, Western democracies with advanced technological infrastructures. Second, research colleagues may be overrepresented due to snowball recruitment through the authors' own social media accounts. Third, although, unfortunately, retrospective sampling was restricted by the upper word limit allowed in Google searches, it is not inconceivable that the selected hashtags, terms, and languages may have skewed the results. Fourth, codings were necessarily subjective. While evaluative codings may be verified to control for experimenter bias, many limitations are inherent to anonymous crowdsourcing (Hale et al., 2021). Fifth, because the nine emotion prompts were derived from author consensus rather than any particular theoretical framework, future research may need to reassess the labels. Sixth and finally, our coding scheme makes no attempt to distinguish between truly music-structural and extra-musical features and emotions. Whenever humor, patriotism, conflict, or togetherness are identified, for example, users may need to reinterpret and/or reclassify database entries to suit their individualized needs.

Future researchers may develop this database into a systematic typology of coronamusic, identifying and characterizing key subgenres like health songs, splitscreen performances, contrafacta, and original coronasongs. Its direct and derived content may become subject to musical, acoustical, video-based, or text-based analysis to answer questions about nostalgia (Yeung, 2020), humor (Bischetti et al., 2021), positive sentiment (Bhat et al., 2020), alongside other pertinent topics. Teachers and historians may draw examples from this resource.

Capitalizing on ingrained psychological coping mechanisms (Fink et al., 2021), the coronamusic phenomenon of 2020 illustrates how humans use music to cope through societal crises. These processes may underlie the sociocultural evolution of music itself (Savage, 2019). Since the world was less interconnected and digitalized during prior wars and pandemics, knowledge gained through coronamusic research may be novel and help inform policy-making in human health and well-being.

## Data Availability Statement

The datasets presented in this study can be found in online repositories. The names of the repositories and accession number(s) can be found below: OSF (version number 20210519): https://osf.io/y7z28/; Github (version number 20210519): https://github.com/dana-and-monsters/corona-music-database; ShinyApp: https://dana-and-monsters.shinyapps.io/corona-music-database/.

## Author Contributions

NH conceived of the project, launched crowdsourcing, obtained permissions for data handling, produced the figures, and wrote the initial draft of the manuscript. JT performed a very large amount of the database coding and resolution of coding conflicts. DS created the ShinyApp and contributed to figures. JB created the Python script for retrospective sampling. All authors contributed substantially to research design, coding scheme, content coding, data curation, revision of the initial draft, and approval of the final version of the manuscript.

## Conflict of Interest

The authors declare that the research was conducted in the absence of any commercial or financial relationships that could be construed as a potential conflict of interest.

## Footnotes

^1^Germany (15) later exceeded the ≥15-video threshold after quality checks but was not included as target country for retrospective sampling.
